# Carbonell-Bonafé Component Separation Technique for Giant Recurrent Ventral Hernia With Loss of Domain: A Case Report

**DOI:** 10.7759/cureus.89182

**Published:** 2025-07-31

**Authors:** Pedro Cardenas Cruz, Felipe de Jesus Magaña Torres, Carlos Oswaldo Parra Andrade

**Affiliations:** 1 Surgery, Hospital General de Zona No. 33, Instituto Mexicano del Seguro Social, Bahia de Banderas, MEX; 2 Surgery, Instituto Mexicano del Seguro Social, Guadalajara, MEX; 3 Surgery, Hospital General de Zona 1, Instituto Mexicano del Seguro Social, Tepic, MEX

**Keywords:** abdominal wall surgery, carbonell-bonafé technique, component separation, giant ventral hernia, pneumoperitoneum

## Abstract

Complex ventral hernias pose a significant surgical challenge, particularly those with large defects or loss of domain. Traditional techniques may be associated with high complication rates or insufficient defect coverage. The modified Carbonell-Bonafé anatomical component separation (ACS) technique offers an innovative approach to achieve tension-free closure and functional abdominal wall reconstruction. We report the case of a 44-year-old woman with a giant recurrent ventral hernia (European Hernia Society (EHS) classification M2-4 W3 R6), a history of 11 previous abdominal surgeries, and a 16 × 18 cm defect confirmed on computed tomography (CT). A two-stage surgical approach was chosen: preoperative progressive pneumoperitoneum followed by hernia repair using mesh and the modified Carbonell-Bonafé ACS technique. This method allowed tension-free closure with minimal soft tissue disruption. The patient recovered uneventfully, and imaging at four months confirmed no recurrence. The Carbonell-Bonafé technique was selected due to its advantages in preserving perforator vessels, minimizing wound complications, and allowing safe closure of large and recurrent hernias. Its incorporation of prosthetic reinforcement and preoperative pneumoperitoneum makes it especially valuable for complex cases. This report illustrates the technique's effectiveness and educational relevance for surgeons managing similar challenging scenarios. This case highlights the clinical utility of the modified Carbonell-Bonafé, which represents a safe and effective tool for reconstructing complex abdominal wall defects, expanding surgical options for giant and recurrent ventral hernias. This approach minimizes wound complications and preserves skin vascularization, enhancing functional recovery compared to traditional methods.

## Introduction

Ventral hernias are a common complication following abdominal surgeries, with an estimated incidence of up to 20% after midline laparotomy [1]. Among these, complex ventral hernias, those involving large defects (>10 cm), loss of domain, or multiple recurrences, represent a smaller yet significant subset, comprising approximately 10-15% of all ventral hernia cases [2]. Loss of domain is particularly rare and challenging, often defined radiologically when the volume of herniated contents exceeds 20% of the total abdominal volume [2,3].

The repair of complex ventral hernias represents a significant surgical challenge, particularly in cases involving large defects or loss of domain [4-6]. The component separation technique (CST) is commonly used for the closure of large abdominal wall defects, including giant ventral hernias or those with loss of domain, achieving tension-free closure and anatomical-functional reconstruction [5]. The anatomical component separation (ACS) technique, as modified by Carbonell-Bonafé, is an innovative surgical strategy developed to address complex and catastrophic abdominal wall defects. This approach involves precise separation of the abdominal wall muscular components, allowing for a more effective and tension-free closure of defects larger than 12 cm, recurrent hernias, or cases with loss of domain [4,6]. This report presents a clinical case successfully treated using this technique.

## Case presentation

We present the case of a 44-year-old female patient from Mexico City, a homemaker by occupation. Her medical history included type 2 diabetes mellitus diagnosed four months prior, managed with metformin. Surgical history included 11 previous abdominal surgeries: six for ventral hernia repair, the most recent one performed one year earlier; three cesarean sections, the last one two years prior; and a hysterectomy due to uterine fibroids performed one year ago.

**Figure 1:**
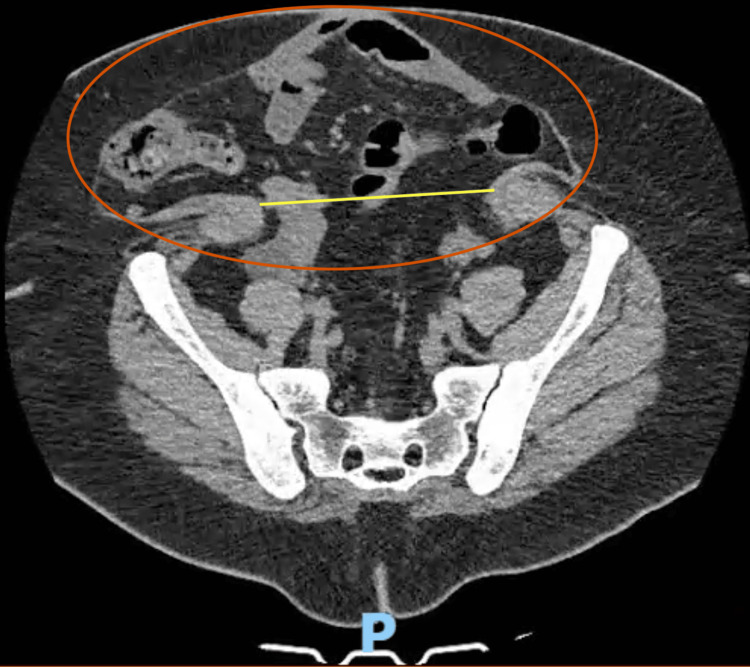
CT scan showing hernia sac measuring 28 x 11 x 25 cm (red circle), with a lateral-to-lateral wall defect measuring 16 x 18 cm (yellow line) CT: computed tomography

The patient was referred to the general surgery department from primary care due to progressive abdominal enlargement, without any associated symptoms such as pain, bowel obstruction, or skin changes. She reported no acute complaints and remained clinically stable. However, the significant abdominal deformity and history of multiple prior surgeries prompted elective evaluation. Physical examination and subsequent imaging confirmed the diagnosis of a giant ventral hernia, classified as M2-4 W3 R6 according to the European Hernia Society (EHS) classification [1]. Imaging evaluation with computed tomography (CT) confirmed discontinuity of the anterior midline in the mesogastric and hypogastric regions, with herniation of the small intestine, ascending colon, right transverse colon, and sigmoid colon. The hernia sac measured 28 × 11 × 25 cm, and the abdominal wall defect was 16 × 18 cm in the transverse and craniocaudal dimensions (Figure 1).

**Figure 2:**
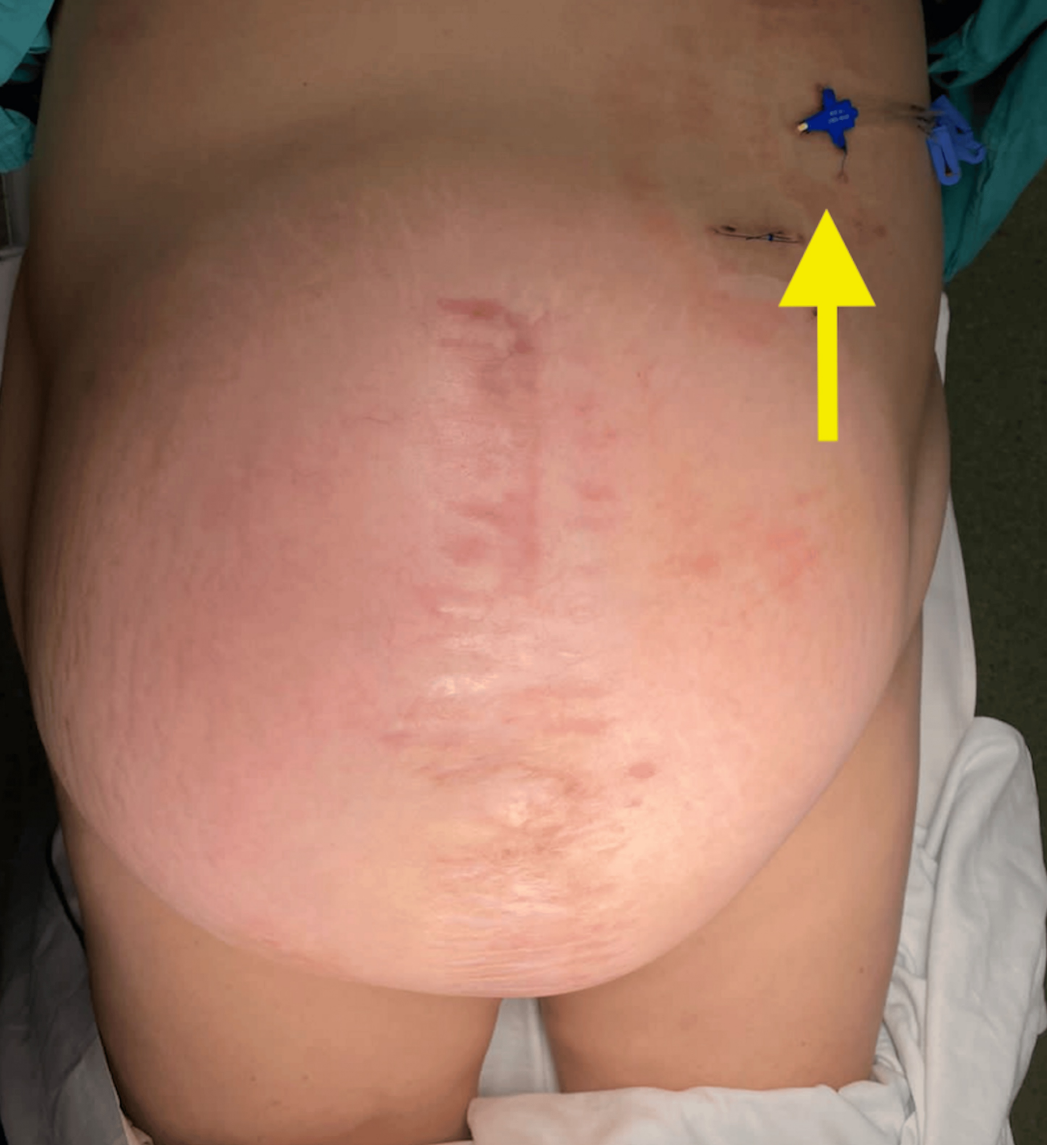
Placement of pneumoperitoneum catheter (yellow arrow)

**Figure 3:**
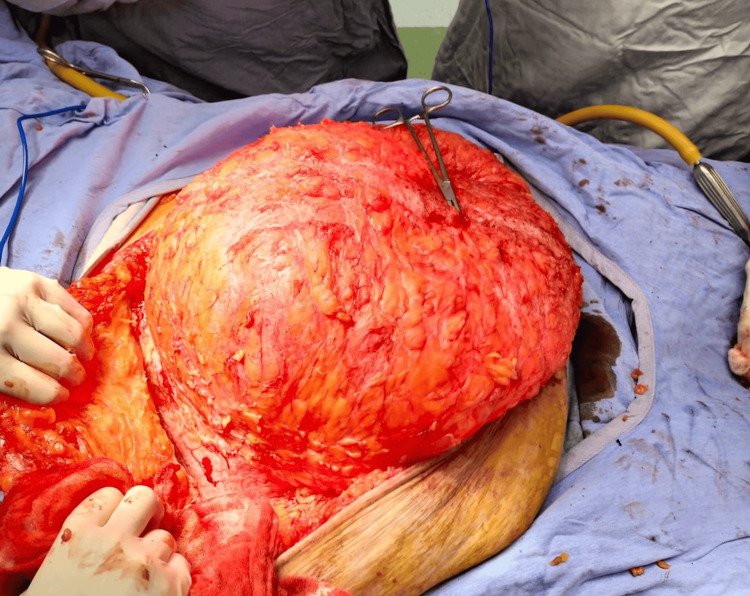
Dissection of the hernia defect

**Figure 4:**
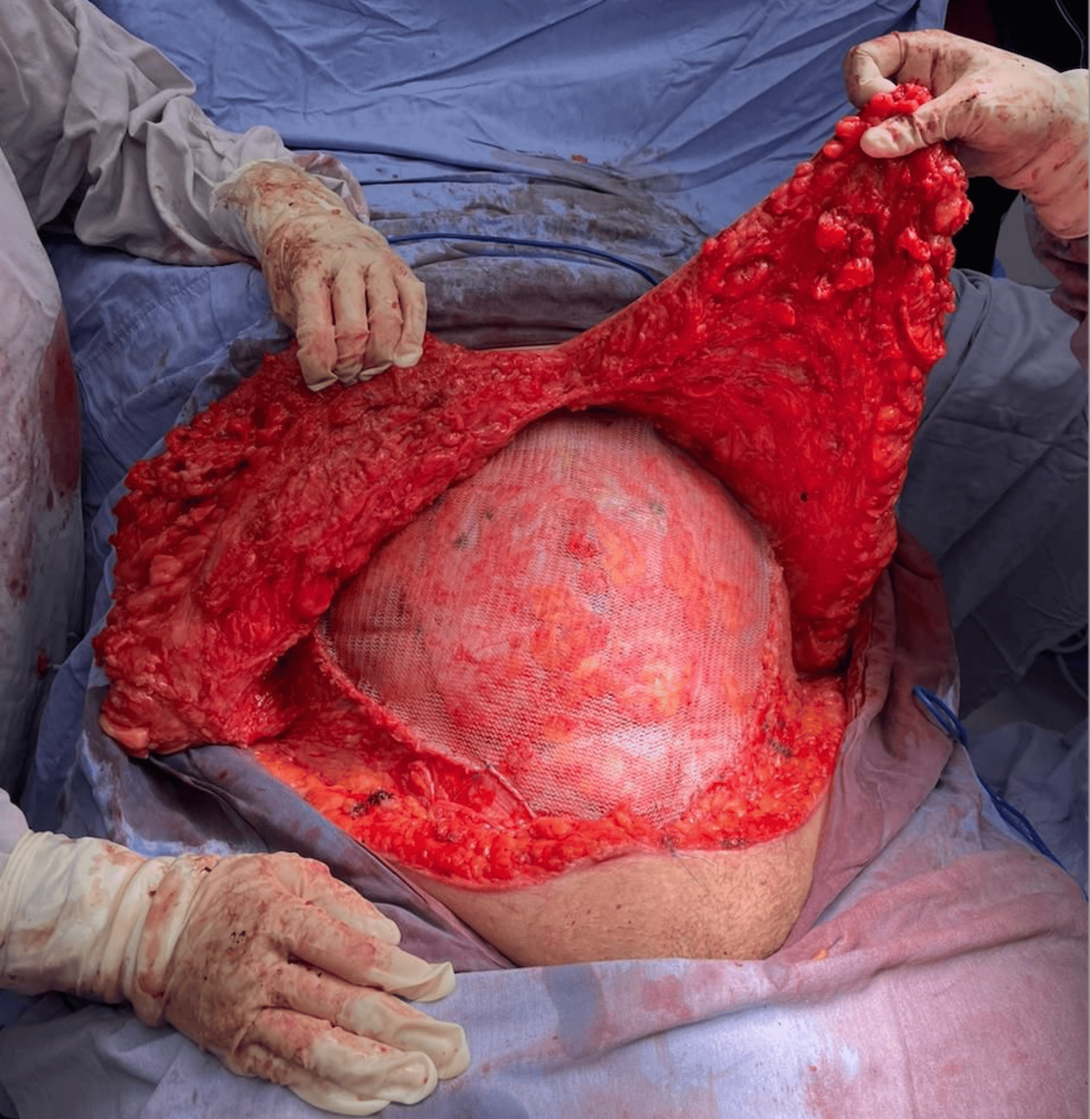
Mesh placement

**Figure 5:**
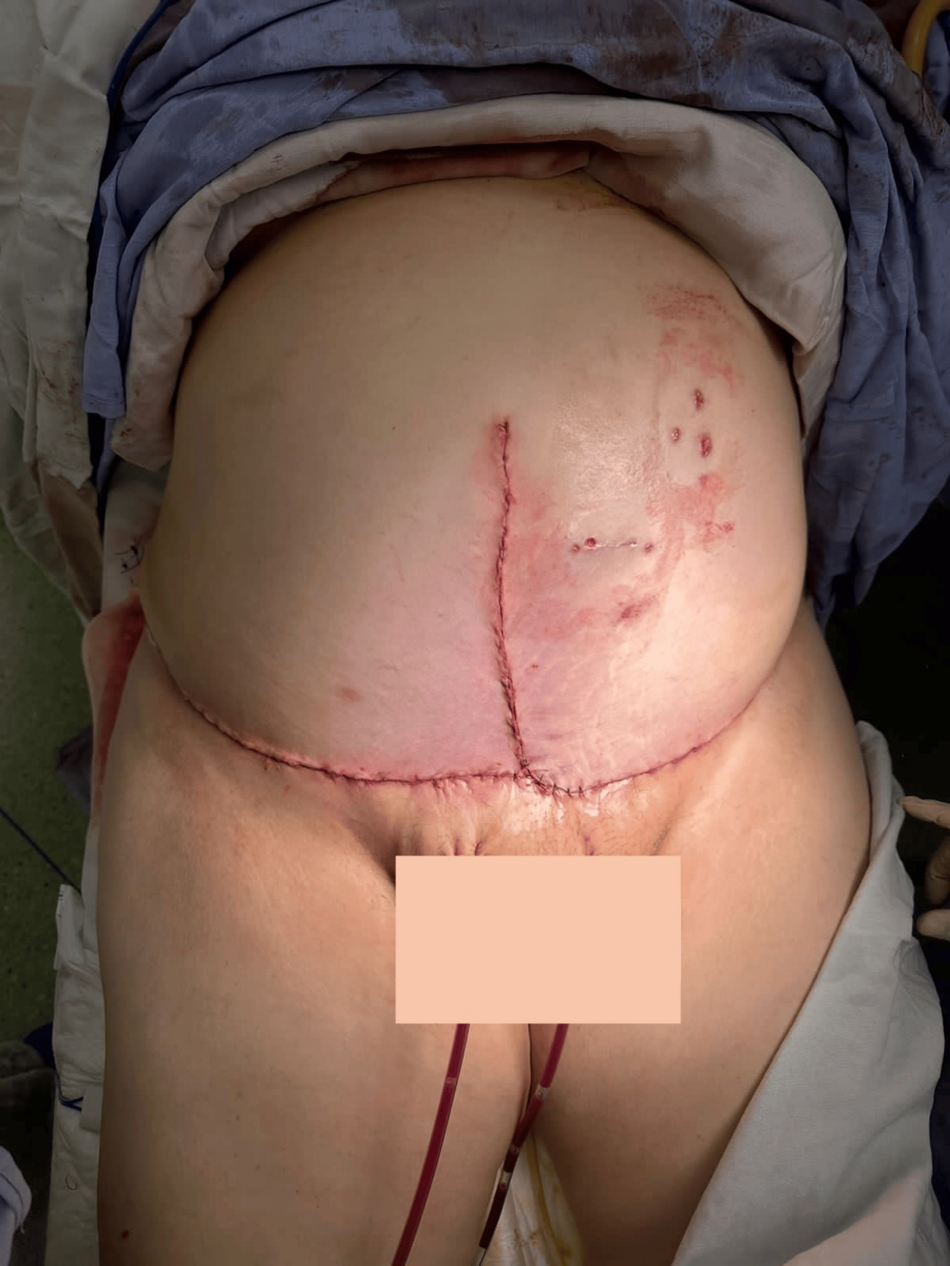
Final tissue closure and skin approximation

**Figure 6:**
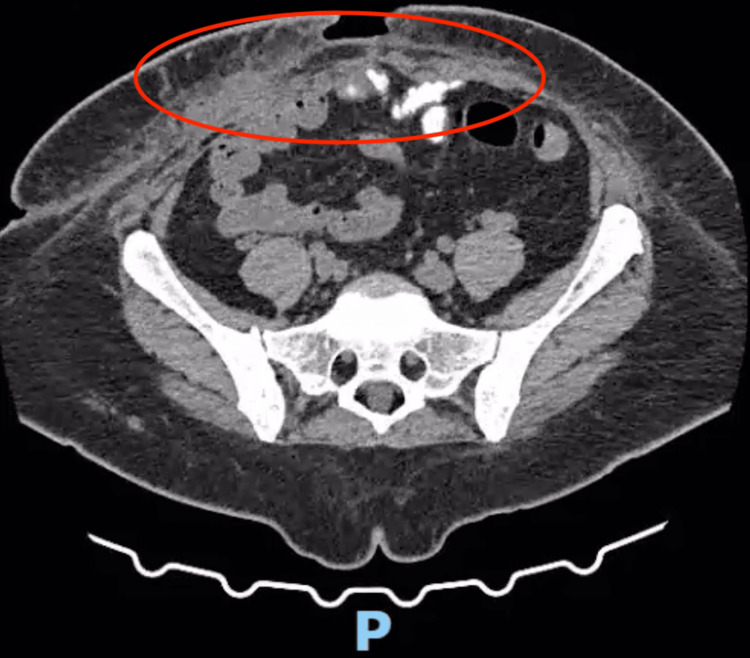
CT scan one year after surgery no wall defect (red circle) CT: computed tomography

A two-stage surgical approach was planned. In the first stage, a preoperative pneumoperitoneum catheter was placed and insufflated with 1000 cc (Figure 2), as recommended in the Carbonell-Bonafé technique for managing giant or loss-of-domain hernias, to facilitate safe abdominal content reintegration and minimize closure tension [4]. After two weeks, the second stage was carried out, consisting of ventral hernia repair with mesh and anterior component separation using the Carbonell-Bonafé technique, plus dermolipectomy. The patient was supine position under general anesthesia. Aseptic preparation and antisepsis of the surgical site were performed, and sterile drapes were placed. A midline supra- and infraumbilical incision was made, and the hernia defect was dissected. The hernia sac was identified and freed up to the edges of the defect (Figure 3). Bilateral dissection of the external oblique fascia was carried out up to the midaxillary line. A 35 × 30 cm polypropylene mesh was placed and secured with continuous Prolene 0 sutures to the borders of the external oblique (Figure 4). A tubular drain was placed over the mesh, hemostasis was achieved, and Blake-type drains were placed. Layered wound closure was performed (Figure 5). Intraoperative findings include a giant ventral hernia with loss of domain and a hernia defect measuring 25 cm in transverse length × 20 cm in longitudinal length. The patient showed a favorable postoperative evolution, was discharged without complications, and continued follow-up on an outpatient basis. A CT scan performed four months after surgery showed no signs of recurrence (Figure 6). At one-year follow-up, the patient continued to show no evidence of hernia recurrence on clinical evaluation and remained asymptomatic with good clinical progression and improved quality of life. 

## Discussion

The anterior abdominal wall is composed of several layers, including skin, fat, fascia, muscle, and peritoneum. The arrangement of these layers varies depending on the perpendicular location to the abdomen. Approximately halfway between the umbilicus and the pubic symphysis lies an imaginary line called the arcuate line. At this point, the abdominal layers relative to the rectus muscle change orientation. Above the arcuate line, the fascia of the internal oblique aponeurosis envelops the rectus muscle. The external oblique aponeurosis always lies anterior to the internal oblique aponeurosis, and the transversus abdominis aponeurosis lies posterior to it. However, below the arcuate line, the three aponeurotic layers move anterior to the rectus muscle, which is no longer enveloped. Instead, the only fascial layer beneath the rectus is the transversalis fascia, which is separated from the transversus abdominis aponeurosis [2].

The EHS classification groups all ventral hernias larger than 10 cm into a common category (W3) [1,3]. Giant ventral hernias can develop after abdominal surgery but may also arise spontaneously, for example, from umbilical or epigastric hernias. Factors predisposing to hernia formation include postoperative infection, poor surgical technique, common risk factors such as smoking, and comorbidities, including diabetes, obesity, and altered collagen metabolism [3,7].

The CST is a valuable approach traditionally used for large abdominal wall defects, such as giant ventral hernias, recurrent hernias, or those with loss of domain. This technique has demonstrated the ability to achieve primary closure of hernia defects through muscular advancement or the placement of prosthetic mesh while maintaining the normal anatomy and physiology of the abdominal wall, thereby improving postoperative outcomes [1].

In the 1970s, Goñi Moreno proposed preoperative pneumoperitoneum to stretch the abdominal muscles, thereby increasing intraabdominal volume and facilitating reduction of hernia contents, which often consist of intestines, thus avoiding resection when possible. This method has shown high efficacy in patients with catastrophic eventrations where reintegration of herniated contents seemed impossible [4,8].

In the 1990s, Ramirez et al. [1,5,8] introduced the CST to allow parietal elongation and muscle closure. Ramirez technique involves dissection and independent mobilization of the abdominal wall muscle layers, mainly separating the external oblique muscle from the internal oblique and the rectus muscle from its posterior sheath. This allows medial advancement of each muscle component to close large defects without tension, restoring abdominal wall anatomy and function without the need for distant flaps or synthetic meshes. However, it has been associated with higher wound complication rates, such as infection and skin necrosis, due to the extensive subcutaneous dissection required [3,5,6].

Although the Ramirez CST has been widely used for large abdominal wall defects, it is associated with considerable wound complications due to extensive subcutaneous dissection, which can lead to infections and skin necrosis [3,5,6]. The modified Carbonell-Bonafé technique addresses these issues by preserving perforating vessels and minimizing tissue trauma, resulting in lower morbidity and better postoperative outcomes [1,4]. Furthermore, the incorporation of prosthetic reinforcement and preoperative progressive pneumoperitoneum optimizes abdominal wall closure and reduces tension, thereby improving functional recovery [4,6,8]. Comparative studies, such as that by Toma et al. [6], support the superiority of posterior CSTs with transversus abdominis release in selected cases, emphasizing the evolving nature of reconstructive approaches for complex hernias.

The Carbonell-Bonafé modified anatomical CST has proven to be an effective and safe strategy for managing complex midline defects, whether infraumbilical or supraumbilical, especially those larger than 10-12 cm, recurrent, or with loss of domain [4,5,9]. Unlike the original Ramirez method, this technique incorporates prosthetic reinforcement of the abdominal wall to minimize closure tension [4,5]. Additionally, it significantly reduces subcutaneous dissection and preserves skin vascularization, resulting in a lower incidence of wound complications such as infections or skin necrosis [1,4]. Another distinctive feature is the inclusion of new muscle insertions and the use of preoperative measures such as progressive pneumoperitoneum, which allow better patient preparation and facilitate reintegration of hernia contents, thereby optimizing postoperative outcomes [4,6,8,9].

A prospective study published by Carbonell et al. [4] included 100 patients and showed low postoperative morbidity, absence of recurrences, and rapid functional recovery, supporting the clinical utility of this technique.

The Carbonell-Bonafé technique offers specific advantages over other methods. Compared to the original Ramirez anterior component separation, it reduces subcutaneous dissection and preserves perforator vessels, decreasing the risk of wound complications. In contrast to posterior separation with transversus abdominis release (TAR), which requires deeper anatomical dissection and higher technical demand, the Carbonell-Bonafé approach maintains an effective balance between anatomical restoration and surgical simplicity. Although minimally invasive techniques have shown lower wound morbidity, they are not always available or feasible, particularly in resource-limited settings. Our case supports the safety and effectiveness of this technique, particularly in large, recurrent hernias with domain loss.

While the Carbonell-Bonafé technique offers numerous advantages in complex ventral hernia repair, it is not without potential complications. These may include seroma formation, mesh infection, postoperative hematoma, and, in rare cases, recurrence, particularly in patients with poor wound healing or significant comorbidities [4,9]. However, in our patient, no postoperative complications were observed.

In the present case, no intraoperative or postoperative complications were observed. The use of preoperative pneumoperitoneum effectively increased the abdominal domain, allowing successful reintegration of hernia contents without signs of intraabdominal hypertension. The modified Carbonell-Bonafé technique permitted a tension-free closure with preservation of skin vascularization, and no signs of wound infection, dehiscence, or recurrence were noted during the four-month follow-up. These results are consistent with findings by Carbonell et al., who reported low complication rates and no recurrences in their initial series of 100 patients using this technique [4]. While long-term outcomes beyond one year remain to be evaluated in our patient, early results are promising and support the efficacy and safety of this anatomical approach in complex ventral hernia repair.

## Conclusions

The modified Carbonell-Bonafé ACS technique represents a valuable and effective surgical option for the reconstruction of complex ventral hernias, particularly those with large defects, recurrences, or loss of domain. Its anatomical approach preserves vascularization, minimizes subcutaneous dissection, and allows for tension-free closure reinforced with mesh, reducing the risk of wound complications and recurrence. In this case, the technique enabled safe reintegration of herniated contents following preoperative pneumoperitoneum and achieved successful abdominal wall reconstruction without intra- or postoperative complications. At four-month follow-up, the patient remained asymptomatic with no signs of recurrence, highlighting the safety and early effectiveness of this method.

Compared to traditional anterior separation techniques such as the Ramirez method, the Carbonell-Bonafé modification reduces morbidity while maintaining efficacy. When posterior or minimally invasive approaches are not feasible, this technique remains a reliable alternative with proven outcomes, as supported by case series in the literature. Further long-term follow-up and broader studies may strengthen the evidence base and help define clear indications for its use, particularly in patients with limited resources or high surgical risk.
